# The Association between Histidine-Rich Glycoprotein rs10770 Genotype and Recurrent Miscarriage in Iranian Women

**DOI:** 10.1155/2024/2501086

**Published:** 2024-04-17

**Authors:** Mahbobeh Latifimehr, Leila Nazari, Ali Asghar Rastegari, Zahra Zamani, Pezhman Fard-Esfahani

**Affiliations:** ^1^Department of Molecular and Cell Biochemistry, Falavarjan Branch, Islamic Azad University, Isfahan, Iran; ^2^Department of Obstetrics and Gynecology Preventative Gynecology Research Center, Shahid Beheshti University of Medical Sciences, Tehran, Iran; ^3^Department of Biochemistry, Pasteur Institute of Iran, Tehran, Iran

## Abstract

**Purpose:**

Recurrent miscarriage (RM) is a significant reproductive concern affecting numerous women globally. Genetic factors are believed to play a crucial role in RM, making the histidine-rich glycoprotein (HRG) gene, a topic of interest due to its potential involvement in angiogenesis. This study is aimed at investigating the association between the HRG rs10770 genotype and RM.

**Method:**

Blood samples were collected from a total of 200 women at the beginning of the study. Subsequently, a comparative analysis was conducted between the blood samples of 100 women with a history of RM (case group) and the blood samples of another 100 healthy women (control group). HRG rs10770 genotyping was performed through polymerase chain reaction restriction-fragment length polymorphism (PCR-RFLP), followed by statistical analysis to evaluate the relationship between HRG rs10770 genotype and RM.

**Results:**

The results indicated a significant statistical difference between the C/C genotype (OR = 3.32, CI: 1.22-9.04, *p* = 0.01) and the C/T genotype (OR = 1.24, CI: 0.67-2.30, *p* = 0.47) in both the case and control groups. Additionally, a significant correlation was observed in the C allelic frequency among RM participants compared to the control group (OR = 1.65, CI: 1.06-2.58, *p* = 0.02).

**Conclusion:**

The study highlights the importance of HRG rs10770 in understanding RM, shedding light on its implications for reproductive health. Furthermore, it became evident that women carrying the homozygous C/C genotype exhibited increased susceptibility to the risk of RM.

## 1. Introduction

Recurrent miscarriage (RM) is defined as experiencing two or more versus three or more miscarriages before reaching the 20th week of pregnancy. Different definitions have been suggested to describe this issue; however, they vary in terms of the number and order of miscarriages [1]. In this study, RM is defined as experiencing two or more consecutive pregnancies that do not result in a live birth.

When a woman has a higher maternal age and has experienced consecutive miscarriages, the likelihood of experiencing a miscarriage might also increase [1]. Primary RM refers to cases in which individuals have a history of RM but have never had a live birth, as all their pregnancies have ended in miscarriage; however, individuals who have experienced at least one successful pregnancy and had a history of RM are categorized as secondary RM cases [2]. Approximately 15% of diagnosed pregnancies result in miscarriage, which can cause significant physical and emotional strain on couples [3]. Many different factors are known to contribute to the occurrence of RM, including autoimmune, anatomical, infective, endocrine, genetic, and environmental factors, thrombophilic defects, and lifestyle [4].

However, the causes of RM remain unknown in 50% of couples [5]. As a result, the remaining cases are labeled as idiopathic [1]. The creation of new blood vessels from existing ones, known as angiogenesis, is necessary for both embryonic development and the processes of implantation [6]. Histidine-rich glycoprotein (HRG) is a regulator of angiogenesis in plasma [7]. HRG is a single-stranded protein produced by the liver with a molecular weight of 75 kDa [8, 9]. HRG gene in humans is located on chromosome 3 at positions q28-q29 and is responsible for producing a protein with 507 amino acids [8]. HRG protein consists of three main sections: (1) two N-terminal regions (N1 and N2), (2) the histidine-rich region (HRR) surrounded by two proline-rich regions (PRR1 and PRR2), and (3) the C-terminal domain [10]. This protein also has four intradomain disulfide bonds, two interdomain disulfide bonds, and six N-glycosylated regions [11]. HRG is a protein that acts as an adapter and can bind to various ligands including heparin [12], tropomyosin [13], plasminogen [9], Zn2+ [14], thrombospondin (TSP) [15], heme [16], fibrinogen [17], complement components [18], phospholipids [19], vasculostatin [20], and immunoglobulin G [21]. Also, HRG is involved in the regulation of many biological processes, including blood coagulation, angiogenesis, fibrinolysis, and the immune system [10]. HRG plays a role in various processes that are crucial for pregnancy; however, the specific role it plays in fertility has remained unknown. HRG can be found in both the reproductive system and the fetus [22]. Additionally, it contributes to the excessive coagulation and imbalance of angiogenesis observed in early preeclampsia [23].

There are at least ten naturally occurring single nucleotide polymorphisms (SNPs) of HRG [24]. HRG C633T SNP has been suggested to potentially be linked to successful implantation and pregnancy [25], and there is a higher occurrence of the T/T genotype in individuals who have experienced early RM [26]. In a SNP, which is called HRG rs10770, a nucleotide (T) is replaced by a C. This replacement changes isoleucine to threonine at amino acid position 180 in the protein.

HRG rs10770 is situated in the cystatin domains, which is the center of the N-terminal domains of HRG. These domains are similar to the N-terminal domain of antithrombin III and are involved in heparin binding. Also, both HRG and antithrombin III have corresponding domains that play a role in heparin binding. The N1 and N2 terminal domains of HRG interact with C1q, IgG, and FcgRI to regulate immune system function [27].

There is currently a lack of research on the role of HRG rs10770 in pregnancy. HRG is known for its role in the proper regulation of angiogenesis during implantation and placental development; therefore, this study is aimed at examining the association between HRG rs10770 and RM.

## 2. Materials and Methods

### 2.1. Subject Collection

This research studied blood samples from two groups of female volunteers who visited specialists at Taleghani Hospital in Tehran between 21 April 2020 and 6 June 2022. The Ethics Committee's approval (IR.IAU.NAJAFABAD.REC.1397.020) was obtained to fulfill the ethical requirements for the study. This study with a case-control study design included two groups, namely, the control and case groups. The PASS 15.0.5 software was utilized to determine the sample size of the study. The results with a 95% confidence interval and 80% power revealed that 100 samples were required for each group to ensure the statistical validity of the study. Since the samples under study were rare, the number of the participants did not exceed the specific number determined by PASS software. Accordingly, the control group for this study consisted of 100 women who were under 35, had given birth to at least two children, and had no previous history of RM. The case group was composed of 100 women who had encountered at least two occurrences of RM in the past. The women experiencing RM were in the age range of 28 to 35 years, and the reason for their RM was not identified. Those patients whose RM was determined to be because of autoimmune, anatomical, infective, endocrine, genetic, and environmental factors, thrombophilic defects, and lifestyle were excluded from the study by the gynecologists and infertility specialists before the commencement of the study. Only those who were determined to have unexplained RM were selected based on the diagnoses of the gynecologists.

### 2.2. Sample Collection and Preparation

A half milliliter of blood was collected from each participant. 200 microliters of this blood was placed on special cards for DNA collection and left to dry for 12 hours. The dried cards were then used to conduct polymerase chain reaction (PCR) testing.

### 2.3. DNA Extraction

Kowsar Biotechnology Company's Washing Buffer was utilized to obtain DNA from DNA storage cards, and the DNA extraction process was done following the guidelines provided by the company. The genotypes of the samples were determined through the use of a polymerase chain reaction restriction-fragment length polymorphism (PCR-RFLP) reaction. Specifically, two primers were designed by Gene Runner (6.0.12) and were then produced by Sinaclon to identify HRG rs10770 gene polymorphism.

The sequence of primers is delineated in Table 1. The length of the product for the forward and reverse primers amplifies a fragment of 376 base pairs (bp).

The materials used in this experimental study were as follow: 15 microliters of Master Mix 2x (Sinaclon, Iran), one microliter from every primer (Sinaclon, Iran), two pieces of DNA extracted from a special DNA storage card, and 13 microliters of deionized distilled water for each sample.

**Figure 1:**
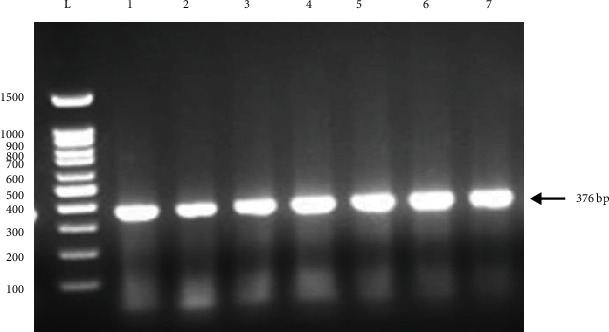
Agarose gel electrophoresis after PCR. L: 100 bp DNA ladder; 1, 2, 3, 4, 5, 6, and 7 show the 376 bp fragment of *HRG gene*.

The reaction mixtures were denatured at 95°C for 5 min, followed by 40 cycles of 95°C for 1 min, 55°C for 1 min, and 72°C for 1 min, with a final elongation at 72°C for 7 min (PeQ Lab, United Kingdom). The PCR products were separated using electrophoresis 2.5% agarose gel (Dena Gene Tajhiz Midi HD, Iran), stained by Green Viewer DNA (Cooperative Mahan Shimi Co, Iran), and visualized by gel documentation system (EBOX VX5, France). According to Figure 1, band 376 bp corresponds to PCR product mentioned in Table 1.

Then, the PCR product was subjected to enzymatic cleavage under the influence of TaqI restriction enzyme. The RFLP reaction included 20 microliters of PCR solution, one microliter of the enzyme, and three microliters of enzyme buffer, and the final volume was 24 microliters. The samples were incubated at 65°C for 3 hours. Finally, the enzyme-digested products were observed by electrophoresis using 3% agarose gel.

**Figure 2:**
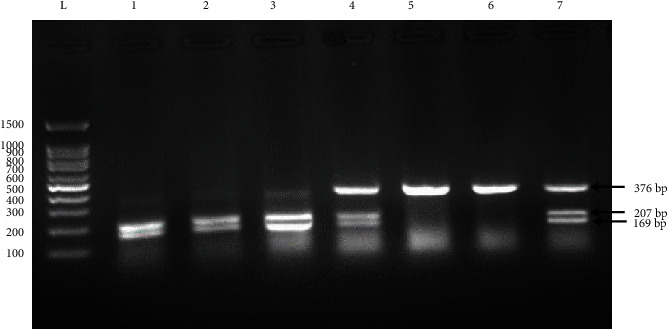
Agarose gel electrophoresis after RFLP. Lanes: L, 100 bp DNA ladder; 1, 2, and 3, homozygous T/T; 4 and 7, heterozygous C/T; 5 and 6, homozygous C/C.

After enzymatic treatment, it was shown that C/C genotype produced only one fragment with a length of 376 bp; C/T genotype produced three fragments with a length of 376, 207, and 169 bp; and T/T genotype produced two fragments with a length of 207 and 169 bp (Figure 2).

### 2.4. Statistical Analysis

The chi-square and odds ratio (OR) tests were carried out using the Statistical Package for Social Sciences (SPSS) software, version 26, to examine the data. The results were reported in terms of *p* value and confidence interval (CI). In this study, *p* < 0.05 was considered statistically significant.

## 3. Results

This study examined whether the HRG rs10770 polymorphism has any statistically significant relationship with RM. The study included 100 participants who had a history of RM and another 100 participants who did not have a history of RM and had at least two successful pregnancies.

The characteristics of the participants including their age range, body mass index (BMI), and genotypes are presented in Table 2. Since conditions like diabetes and metabolic syndrome, which can be associated with higher BMI, may contribute to recurrent miscarriages [28], BMI has been taken into consideration in this study. The study found that out of 100 women with RM, 52 (52%) were homozygous T/T, 17 (17%) were homozygous C/C, and 31 (31%) were heterozygous C/T. In the control group, out of 100 women examined, 61 (61%) were found to have the T/T genotype, 6 (6%) had the C/C genotype, and 33 (33%) had the C/T genotype. The distribution of genotypes showed no statistically significant differences between cases and controls for the T/T (*p* = 0.15) and C/T (*p* = 0.78) genotypes, as determined by the chi-square test. However, there was a significant difference in the distribution of the C/C genotype between cases and controls (*p* = 0.02) according to Fisher's exact test. This suggests a potential association between the C/C genotype and the occurrence of recurrent miscarriage among Iranian women.

To investigate the correlation between genotypes and the occurrence of RM, the OR analysis was conducted. Moreover, the precision of the OR estimate was specified by reporting the confidence interval (CI) measure. The results of the study revealed that the OR for C/C and C/T genotypes were 3.32 CI (1.22-9.04) and 1.24 CI (0.676-2.308), respectively (Table 3). Also, the OR for C allele was 1.65 CI (1.06-2.58). The results of the chi-square test showed that there was a significant difference between the control and case groups in terms of allelic (*p* = 0.02) and genotypic (*p* = 0.01) differences. The OR test showed that women with the C/C genotype were at a higher risk of RM.

## 4. Discussion

The present study examined the relationship between HRG 10770 SNP and RM using the RFLP technique. The results of the chi-square test showed that there was a significant difference between the control and case groups in terms of allelic (*p* = 0.02) and genotypic (*p* = 0.04) differences. The OR test results also indicated that women with the C/C genotype were at a higher risk of RM. Also, the results of the study indicated that there was a positive correlation between the HRG 10770 SNP polymorphism and the likelihood of RM in terms of both allelic and genotypic variations.

Establishing pregnancy requires adequate regulation of angiogenesis, coagulation, and regulation of immune pathways. The precise coordination of these processes is complex, and defects in any of the factors mentioned may cause pregnancy failure [1]. The microarray analyses of endometrial biopsies and decidual samples among women with RM have demonstrated that genes associated with various functional areas were not adequately regulated. The most significant genes found to be affected were those that regulate cell adhesion, cell migration, and angiogenesis [29, 30].

Additionally, the studies that have investigated the relationship between gene polymorphisms coding and the mediators of angiogenesis have shown that there have been positive associations between gene polymorphisms coding and angiogenesis [6, 31].

There have been accounts of how HRG can either improve or impede angiogenesis, affecting both the specific and broader functions of cells. HRG is known to be an important protein in early pregnancy, and former studies have revealed the existence of a positive relationship between HRG in the placenta and the risk of preeclampsia during pregnancy [32]. Moreover, a relationship between HRG C633T SNP and pregnancy outcome has been found among cases undergoing *in vitro* fertilization [25].

The investigation of the effect of HRG C633T polymorphism on the increased risk of pregnancy blood pressure disorder has shown that the presence of the T allele plays a role in increasing the risk of pregnancy blood pressure disorder [23]. In the current study, the SNP HRG rs10770 was related to the mutation in the N-terminal region of HRG. In this mutation, one nucleotide (T) is replaced by C, which changes isoleucine to threonine at amino acid position 180 in the protein. This mutation has an important role in antiangiogenesis [11] and angiogenesis [32]. HRG is not considered a factor for angiogenesis; however, it can affect angiogenesis in different ways [11].

HRG inhibits thrombospondins 1 and 2 and causes interference in the binding of thrombospondin and its receptor CD36 through the second N-terminal; therefore, it increases angiogenesis [33]. The substitution of isoleucine with threonine might lead to an impaired interaction between CD36, TSP, and HRG.CD36 receptor is expressed in the endometrium of the human uterine lining; thus, women who are heterozygous or homozygous for the HRG 10770 C allele might have dysregulation of angiogenesis which leads to implantation and placental defects.

HRG inhibits the activity of heparin sulfate by interacting with heparin sulfate on the surface of endothelial cells. Heparin sulfate acts as a coreceptor for the differentiation of fibroblast growth factors (FGFs). While heparin sulfate interacts on the cell surface, FGFs might bind to its receptor, activate the receptor, and exert the biological function of FGFs. HRGP can compete with FGFs in binding to cell surface heparin sulfate, thereby reducing the effectiveness of FGFs in activating FGF receptors. This inhibition blocks FGF-dependent proliferative activities; however, this inhibition is not limited to angiogenesis [11, 21].

HRGP may cover the heparanase cleavage sites on extracellular matrix's heparan sulfate, thereby preventing the discharge of angiogenesis growth factors (such as FGFs) from the ECM through heparanase-mediated processes. Heparan sulfate plays a crucial role in both the extracellular matrix and the foundational layer of blood vessels, acting as a protective barrier against extravasation. The cutting of heparan sulfate through heparanase can aid in breaking down the ECM and basal layer, making it easier for cells to move around. Likewise, this inhibition is not confined to angiogenesis [11]. A change in the structure of HRG can disrupt this pathway; as a result, HRG cannot inhibit the activity of heparin sulfate and may affect angiogenesis and cause defects in implantation and placenta formation [1].

## 5. Conclusion

In this research, by examining the relationship between rs10770 HRG genotype and RM, it was determined that there was a statistically significant relationship between rs10770 HRG genotype and RM, which may be due to implantation and placental disorders. However, to confirm the results of this study, it is imperative to conduct further studies with a greater sample size.

## 6. Research Limitations

The number of people studied in this research was limited. To determine the genetic pattern, only rs10770 HRG polymorphism was used. The laboratory method for determining the genetic pattern was limited to RFLP.

## Figures and Tables

**Table 1:** Names, sequence, and PCR product of primers.

Names of primers	Sequence of primers	PCR product (bp)
rs10770-F-TaqI	5′-CCAAGCCACCATTAACATTTCC-3′	376
rs10770-R-TaqI	5′-ACCTGCTGCCTGTCTTTTATGG-3′	376

**Table 2:** Characteristics of the studied subjects, including the case and control groups.

	Cases (*n* = 100)	Controls (*n* = 100)	Test type	*p* value
Age (years)	31.56 ± 3.10	29.45 ± 3.34	*t*-test	0.00
BMI (kg/m^2^)	24.59 ± 2.98	25.07 ± 3.19		0.26
T/T	52 (52%)	61 (61%)	Chi-square	0.15
C/T	31 (31%)	33 (33%)		0.78
C/C	17 (17%)	6 (6%)	Fisher's exact test	0.02

**Table 3:** OR values of genotypes and alleles.

	Odds ratio (95% CI)	*p*
Genotype		
T/T	Ref	
C/T	1.24 (0.67-2.30)	0.47
C/C	3.32 (1.22-9.04)	0.01
Allele		
T	Ref	
C	1.65 (1.06-2.58)	0.02

## Data Availability

Data will be made available on request.
